# The *cdx* Genes and Retinoic Acid Control the Positioning and Segmentation of the Zebrafish Pronephros

**DOI:** 10.1371/journal.pgen.0030189

**Published:** 2007-10-19

**Authors:** Rebecca A Wingert, Rori Selleck, Jing Yu, Huai-Dong Song, Zhu Chen, Anhua Song, Yi Zhou, Bernard Thisse, Christine Thisse, Andrew P McMahon, Alan J Davidson

**Affiliations:** 1 Center for Regenerative Medicine, Massachusetts General Hospital, Boston, Massachusetts, United States of America; 2 Department of Molecular and Cellular Biology, Harvard University, Cambridge, Massachusetts, United States of America; 3 Shanghai Institute of Hematology, Rui-Jin Hospital, Shanghai Second Medical University, Shanghai, China; 4 Department of Medicine, Division of Hematology/Oncology, Children's Hospital, Boston, Massachusetts, United States of America; 5 Dana-Farber Cancer Institute, Boston, Massachusetts, United States of America; 6 Institut de Génétique et de Biologie Moléculaire et Cellulaire (IGBMC), Illkirch, France; University of Pennsylvania School of Medicine, United States of America

## Abstract

Kidney function depends on the nephron, which comprises a blood filter, a tubule that is subdivided into functionally distinct segments, and a collecting duct. How these regions arise during development is poorly understood. The zebrafish pronephros consists of two linear nephrons that develop from the intermediate mesoderm along the length of the trunk. Here we show that, contrary to current dogma, these nephrons possess multiple proximal and distal tubule domains that resemble the organization of the mammalian nephron. We examined whether pronephric segmentation is mediated by retinoic acid (RA) and the *caudal* (*cdx*) transcription factors, which are known regulators of segmental identity during development. Inhibition of RA signaling resulted in a loss of the proximal segments and an expansion of the distal segments, while exogenous RA treatment induced proximal segment fates at the expense of distal fates. Loss of *cdx* function caused abrogation of distal segments, a posterior shift in the position of the pronephros, and alterations in the expression boundaries of *raldh2* and *cyp26a1*, which encode enzymes that synthesize and degrade RA, respectively. These results suggest that the *cdx* genes act to localize the activity of RA along the axis, thereby determining where the pronephros forms. Consistent with this, the pronephric-positioning defect and the loss of distal tubule fate were rescued in embryos doubly-deficient for *cdx* and RA. These findings reveal a novel link between the RA and *cdx* pathways and provide a model for how pronephric nephrons are segmented and positioned along the embryonic axis.

## Author Summary

In the kidney, structures known as nephrons are responsible for collecting metabolic waste. Nephrons are composed of a blood filter (glomerulus) followed by a series of specialized tubule regions, or segments, which recover solutes such as salts, and finally terminate with a collecting duct. The genetic mechanisms that establish nephron segmentation in mammals have been a challenge to study because of the kidney's complex organogenesis. The zebrafish embryonic kidney (pronephros) contains two nephrons, previously thought to consist of a glomerulus, short tubule, and long stretch of duct. In this study, we have redefined the anatomy of the zebrafish pronephros and shown that the duct is actually subdivided into distinct tubule segments that are analogous to the proximal and distal segments found in mammalian nephrons. Next, we used the zebrafish pronephros to investigate how nephron segmentation occurs. We found that retinoic acid (RA) induces proximal pronephros segments and represses distal segment fates. Further, we found that the *caudal* (*cdx*) transcription factors direct the anteroposterior location of pronephric progenitors by regulating the site of RA production. Taken together, these results reveal that a *cdx*-RA pathway plays a key role in both establishing where the pronephros forms along the embryonic axis as well as its segmentation pattern.

## Introduction

The kidney eliminates metabolic waste in the body using highly specialized structures called nephrons. Individual nephrons are composed of a blood filter (renal corpuscle), a tubule that recovers or secretes solutes, and a collecting duct [1]. The renal corpuscle contains epithelial cells called podocytes that form the slit-diaphragm filtration barrier and allow collection of substances from the blood [2]. In a number of vertebrate species, including some mammals, the renal corpuscle is connected to the tubule by a short stretch of ciliated epithelium called the neck segment that guides filtrate entry into the tubule [3–5]. The mammalian nephron tubule is subdivided into a series of proximal and distal segments connected to a collecting duct [1,6]. The polarized epithelial cells in the tubule segments have a unique ultrastructure and express a select cohort of solute transporters [1]. Thus, each segment is functionally distinct and performs the transport of particular solutes that are required for proper renal function.

In higher vertebrates, three kidneys of increasing complexity arise sequentially from the intermediate mesoderm (IM): the pronephros, the mesonephros, and the metanephros [7]. The pronephros and mesonephros degenerate in succession, with the metanephros serving as the adult kidney. Lower vertebrates, such as fish and amphibians, develop a pronephros during embryonic stages, and then form a mesonephros that will be used throughout their adult life [8–10]. Each of these kidneys contains the nephron as its basic functional unit [8].

To date, much of our knowledge of kidney development has come from gene-targeting studies in the mouse [7,11,12]. These experiments have identified a number of genes that play essential roles in the early stages of metanephros development, but there is a limited understanding of the molecular pathways governing the later stages of kidney ontogeny, when individual nephrons form and become segmented [7].

The zebrafish is an ideal genetic and developmental model system for dissecting the molecular mechanisms of nephron formation because of the anatomical simplicity of the pronephros, which contains two nephrons as opposed to the thousands of nephrons in a mammalian metanephros [9]. During zebrafish development, bilateral stripes of IM lying on either side of the trunk undergo a mesenchymal-to-epithelial transition to form the pair of pronephric nephrons. The anteriormost renal progenitors differentiate into podocytes, which migrate medially and fuse at the midline to form a single renal corpuscle. The nephrons also fuse posteriorly at the cloaca to form a shared exitway. From a functional standpoint, these pronephric nephrons have been thought to consist of three parts: (1) the blood-filtering renal corpuscle, (2) a very short tubule region that transports solutes, and (3) long pronephric ducts that convey the resulting waste to the cloaca [9]. Contrary to this model, recent studies have suggested that the ‘duct' region possesses regional segmentation, based on the restricted expression boundaries of solute transporter orthologues known to be expressed in the tubule segments of metanephric nephrons. For example, a rostral stretch of the pronephric duct expresses the endocytic receptor *megalin* (*lrp2*) [13] and the sodium bicarbonate transporter *NBC1* (*slc4a4*) [14], which are expressed in the proximal tubule in mammals. These reports raise the possibility that portions of the pronephros considered to be duct might in fact be tubule, thus suggesting that the organization of the zebrafish pronephros is more complex than previously appreciated. However, a complete model of the molecular anatomy of the zebrafish pronephros and whether there is a functional correlation to the segments of the mammalian nephron remain unclear. Furthermore, the pathway(s) directing segmentation of the pronephros along the embryonic axis are unknown.

Numerous factors are known to control segmental patterning along the anterior-posterior (A-P) axis during vertebrate development and thus provide candidate pathways that might act to establish pronephros segmentation. Retinoic acid (RA) signaling is vital for directing the A-P regionalization of tissues deriving from all three germ layers, such as the hindbrain, paraxial mesoderm, and gut [15–19]. Control of RA production via retinaldehyde dehydrogenase (RALDH) synthesizing enzymes [20] and the degradation of RA via the CYP26 catabolizing enzymes establishes both the location and timing of RA signaling [21,22]. In addition to RA, the *caudal* (*cdx*) transcription factors (*Cdx1*, *Cdx2*, and *Cdx4* in mammals and *cdx1a* and *cdx4* in zebrafish) are responsible for determining vertebral identity and directing posterior body formation [23–31]. *cdx* genes are known to act as master regulators of the *homeobox* (*hox*) transcription factors [25], and in turn, overlapping domains of *hox* gene expression along the A-P axis are thought to confer segmental identities [32]. In mice, loss of *Cdx* function causes posterior shifts in *Hox* gene expression that are associated with abnormal vertebral patterning, and posterior truncations due to defects in the extension of the embryo axis [23,25–27,33]. Similarly, studies in zebrafish have shown that the loss of *cdx4* function or deficiency of both *cdx1a* and *cdx4* causes shifts in *hox* gene expression domains, a shortened body axis, and altered patterning of the blood, vascular, and neural tissues [24,28–31]. These lines of evidence indicate that the *cdx* genes play essential roles in controlling cell fates along the embryonic axis; however, the molecular mechanisms underlying these effects have not been elucidated [34].

In this study, we undertook a functional genomics approach to identify new markers of the zebrafish pronephros. From this analysis, we found that the pronephros is composed of at least eight regions, including two proximal and two distal tubule segments. We explored how segmental identity is controlled during nephrogenesis by testing the roles of RA signaling and the *cdx* genes. We found that RA is required to induce proximal segment fates and prevent the expansion of distal segment fates, whereas the *cdx* genes are necessary for positioning the pronephros along the embryonic axis. Embryos deficient in *cdx1a* and *cdx4* displayed a posterior shift in the location of the pronephros and formed proximal but not distal nephron segments. The *cdx* genes were found to control the expression boundaries of *raldh2* (*aldh1a2*) and *cyp26a1*, suggesting a model in which the *cdx* pathway influences where the pronephros forms along the body axis by localizing the source of RA, while subsequent RA signaling acts to direct the segmentation of the pronephros.

## Results

### The Zebrafish Pronephros Consists of at Least Eight Discrete Segments

**Figure 1:**
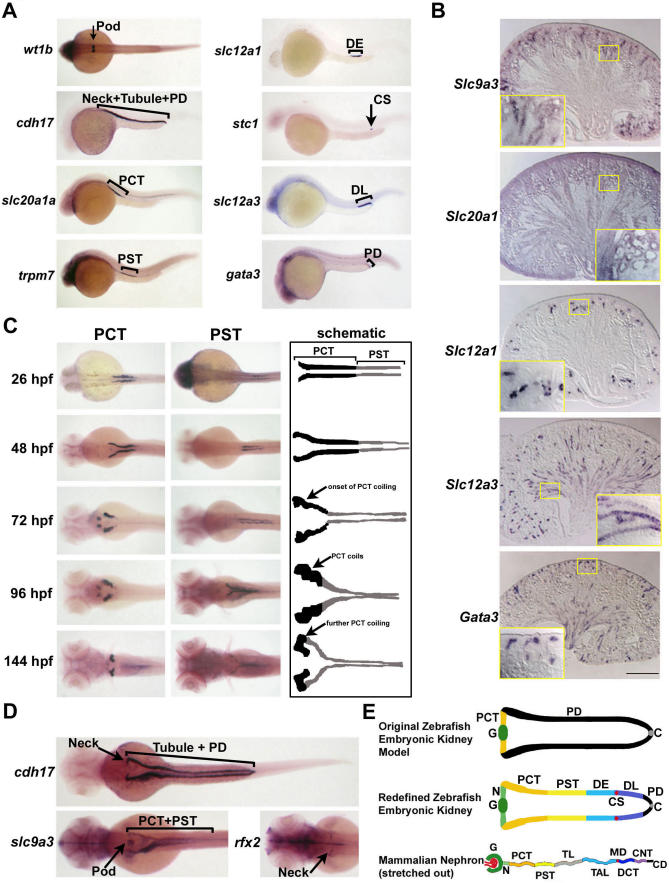
The Zebrafish Pronephric Nephrons Consist of Multiple Tubule Segments Similar to Metanephric Nephrons (A) Whole-mount in situ hybridization of wild-type embryos shows segmentation of each pronephric nephron into podocytes (Pod), neck (N), proximal convoluted tubule (PCT), proximal straight tubule (PST), distal early (DE), corpuscle of Stannius (CS), distal late (DL), and pronephric duct (PD) that fuse to the cloaca (C) (not shown). Lateral views are shown except for dorsal view in top left panel, with anterior to the left. (B) Murine section in situ hybridzation of solute transporters, bar denotes 500 μm. (C) Time course of PCT convolution and adjacent PST, with *slc20a1a* expression used to label the PCT at all stages, PST labeled by *trpm7* expression at 26 hpf, and *slc13a1* expression at 48–144 hpf. (D) Expression of *cdh17*, *slc9a3*, and *rfx2* at 48 hpf in distinct segments of the pronephros. Note expression of *rfx2* in the presumptive ciliated neck segment. (C and D) Dorsal views are shown, with anterior to the left. (E) Schema of the original model of the 48 hpf zebrafish pronephros (top), our redefined model showing segmentation of each nephrons (middle), and a mammalian nephron stretched out to compare segmentation of the metanephric nephron (bottom). Mammalian segments are color coded according to observations of shared solute transporter expressions with respective zebrafish pronephros segments. Additional abbreviations are: G (glomerulus), N (neck), TL (thin limb), TAL (thick ascending limb), MD (macula densa), DCT (distal convoluted tubule), CNT (connecting tubule), and CD (collecting duct).

To gain insight into the molecular mechanisms that control vertebrate renal development, we undertook a functional genomics approach to identify genes expressed in the kidney. We mined two gene collections, one comprising developmentally expressed genes from embryonic zebrafish cDNA libraries [35] and another compiled from an adult zebrafish kidney library [36]. Gene expression patterns were analyzed by whole-mount in situ hybridization using wild-type zebrafish embryos between the 5 somite stage and 144 hours post fertilization (hpf). We identified a number of genes, including 15 solute transporters, that were expressed within specific subregions of the pronephros. In total, eight distinct regions could be visualized, with some genes expressed in more than one region (Figure 1A). Representative examples of region-specific genes include *wt1b*, *slc20a1a*, *trpm7*, *slc12a1*, *stc1*, *slc12a3*, and *gata3*, as compared to expression of *cdh17*, which is found in all tubule and duct progenitors [37] (Figure 1A). We investigated where the mouse or human orthologues of some of these genes are expressed in the mammalian metanephric kidney, and found that many corresponded to segment-specific domains within the nephron. For example, *Slc9a3* is expressed in podocytes, the proximal convoluted segment (PCT) and proximal straight segment (PST) (Figure 1B) [38]. *Slc20a1* is expressed throughout the entire nephron epithelium, although stronger expression was observed in proximal tubule segments (Figure 1B). Transcripts for *Slc13a3* are found in the PST [39], while *Slc12a1* is restricted to the thick ascending limb (TAL) and macula densa (MD) (Figure 1B) [40,41]. *Slc12a3* is expressed in the distal convoluted tubule (DCT) (Figure 1B) [41]. Lastly, *GATA-3* expression specifically marks the collecting ducts (CD) (Figure 1B) [42,43].

Based on this cross-species gene expression comparison, the following identities were assigned to the zebrafish pronephros segments we observed (going from proximal to distal): podocytes (pod), neck (N), PCT, PST, distal early (DE), corpuscle of Stannius (CS), distal late (DL), and the pronephric duct (PD) (Figure 1A and 1E). Our division of PCT and PST within the tubule is based on the observation that the *slc20a1a*-expressing PCT cells undergo morphogenesis from a linear tube into a coiled structure by 5 days post-fertilization (dpf), while the *trpm7-* and *slc13a1*-expressing PST segment maintains a linear structure (Figure 1C). Expression of *trpm7* and *slc13a1* is discontinuous within the PST, an observation that has been shown recently to reflect the presence of two cell types in this region: transporting epithelia and multiciliated cells [44,45]. The renal corpuscle connects to the PCT via a short segment of cells that express the transcription factor *rfx2*, and fails to express almost all of our PCT solute transporters (Figure 1D). As *rfx2* marks ciliated cells and *rfx* genes are essential regulators of ciliogenesis [46,47], we hypothesize that this region corresponds to the ciliated neck segment found in other fish species as well as mammals [3–5]. However, a more detailed analysis is needed to confirm this hypothesis. In addition to the neck segment, *rfx2* expression was also detected in presumptive ciliated cells along the length of the PST and DE segments, as described previously [44,45]. For the distal tubule, we adopted the DE/DL nomenclature used in *Xenopus* [48], although the zebrafish DE appears analogous to the TAL segment in mammals and the DL appears analogous to the mammalian DCT segment according to our gene expression comparison. We included the CS as a discrete segment, as it initially arises from the tubular progenitors within the pronephros, but by 48 hpf, it is located just dorsal to the DE/DL boundary [49,50] (unpublished data). The DL segment expresses *slc12a3* and connects to the cloaca via a short segment that expresses *gata3* and likely represents the PD. Our data are consistent with the notion that the zebrafish pronephric kidney resembles a ‘stretched-out”' mammalian nephron, and suggests that rather than being composed of mostly nephric duct (as currently believed), it is made up of extensive proximal and distal tubule epithelium (Figure 1E).

**Figure 2:**
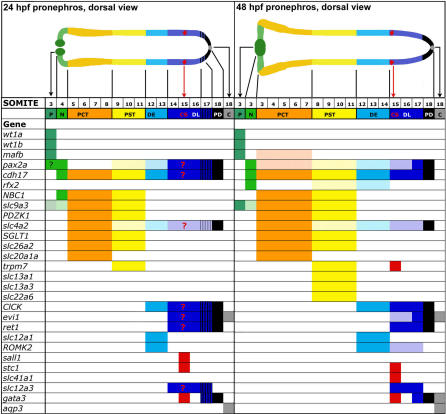
Pronephros Expression of Known Renal Genes and Solute Transporters Isolated Using Functional Genomics The domain of each pronephros-expressed gene was mapped along the embryonic axis according to somite boundaries, using double in situ hybridization with *mhc* to label the myotomes. Solid color bars indicate strong expression, light color bars indicate weak expression, and blue/black stripe bars indicate overlap in the expression domains of DL and PD markers; note that *rfx2* expression at 48 hpf is discontinuous in the PST and DE domains. Schematic anatomy of the pronephros at 24 hpf (top, left) compared to 48 hpf (top, right). The cloaca (C) is not considered a segment of the pronephros, but is shown to depict the terminus of the pronephros.

Between 24 and 48 hpf (the start of blood filtration), the pronephros undergoes significant morphogenesis, including the midline migration of podocytes and the growth/extension of the tubules [9]. In order to better quantitate these morphological changes, as well as to precisely define the anatomical boundaries of each segment, we mapped the expression domains of segment-specific markers relative to the somites by performing double whole-mount in situ hybridization with *myosin heavy chain* (*mhc*) at 24 and 48 hpf (Figures 2 and S1–S4). At 24 hpf, podocyte and neck progenitors are arranged in a slight curve at the level of somite 3–4 with the anterior boundary of the PCT level with somite 5 (Figures 2 and S1). By 48 hpf, the podocyte progenitors have fused at the midline (level with somite 3) with the presumptive neck region forming a lateral extension that connects with the PCT also situated at the level of somite 3 (Figures 2 and S3). During this time, the length of the PCT, PST, and DE segments increased, possibly due to cell division within each segment (Figure 2 and S1–S4). This growth may provide the driving force that is responsible for the shift in the anterior boundary of the PCT from somite 5 to somite 3 between 24 and 48 hpf, and for the coiling morphogenesis of the PCT observed between 72 and 144 hpf (Figure 1C). However, the DL segment did not increase in length between 24 and 48 hpf, indicating that there is not a uniform expansion in all segments during development. During juvenile development (2–3 wk post-fertilization) the DL segment is proportionately larger than the other segments, suggesting that its expansion predominates at later stages of development (unpublished data). Interestingly, at 24 hpf, we observed an overlap of the DL and PD expression domains at the level of somite 17 (Figure 2 and Figure S2). This overlap may indicate the presence of an additional segment (such as a discrete CNT equivalent), though to date we have not discovered any genes expressed solely in this domain.

In addition to mapping the morphological changes that occur between 24 and 48 hpf, we also noted segment specific changes in gene expression patterns during this time. For example, transcripts for the solute transporters *slc13a1* (inorganic sulphate transporter), *slc13a3* (sodium-dicarboxylate carrier), and *slc22a6* (organic anion transporter) were all absent from the PST segment at 24 hpf but were found expressed at 48 hpf (Figures 2 and S3; and unpublished data). Similarly, transcripts for *trpm7* (divalent cation-selective ion channel) and *slc41a1* (Mg 2+ transporter) were not found in cells of the CS at 24 hpf but could be detected at 48 hpf (Figure 2 and unpublished data). The up-regulation of these genes likely reflects the maturation/differentiation of the segment epithelia before the onset of blood filtration, which begins around 40 hpf [51,52]. However, the glomerulus does not fully mature until 4 dpf, based on the size exclusion of different-sized fluorescent dextrans [53]. Further profiling of segment expression patterns at later stages is needed to investigate whether the maturation of the transporting epithelial is also an ongoing process.

### RA Is Essential for Podocyte and Proximal Segment Fates and Inhibits Distal Segment Fates

**Figure 3:**
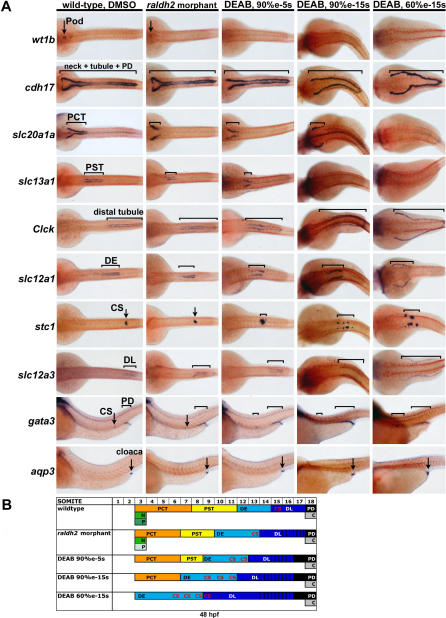
RA Signaling Is Required for Proximal Segment Fates and to Prevent the Expansion of Distal Segment Fates (A) Expression of segment markers and *mhc* at 48 hpf in wild-type embryos and *raldh2* morphants, and wild-type embryos that were incubated with either DMSO (control) or DEAB for indicated times during development, by double whole-mount in situ hybridization. Brackets and arrows indicate expression domains. Abbreviations are: %e (percent epiboly), s (somite stage). Dorsal views are shown, with anterior to the left, with the exception of lateral views for *gata3* and *aqp3* expression. (B) Summary of nephron segmentation with respect to embryo somite number for each DEAB treatment.

We next sought to characterize the developmental pathways that establish the segmentation pattern of the pronephros. Retinoic acid (RA) is essential for the development of numerous tissues during embryogenesis [25]. In vertebrates, a gradient of RA in the upper trunk is responsible for directing the A-P patterning of the hindbrain into segmental compartments [15,54], and gain or loss of RA also affects vertebral identity [25]. Interestingly, RA has been reported to regulate pronephros formation in *Xenopus* [55]. To explore whether RA was needed for pronephros segmentation, we injected wild-type zebrafish embryos with a morpholino to *retinaldehyde dehydrogenase 2* (*raldh2*), which encodes an enzyme required to produce RA [20]. We then examined *raldh2* morphants at 48 hpf with our panel of segment-specific markers, in combination with *mhc* expression to map segment length and location relative to the somites. Embryos deficient in *raldh2* had fewer podocytes, as evidenced by punctate staining of the podocyte markers *wt1b, wt1a,* and *mafb* (Figure 3A and unpublished data). Both proximal tubule segments were slightly shortened, based on the reduced expression domains of *slc20a1a* (PCT) and *trpm7* (PST) (Figure 3). Conversely, the distal tubule was expanded in length, with the *clck* transporter (marking both the DE and DL) expressed in a greater proportion of the *cdh17*-positive pronephric tubule (note that the overall length of *raldh2* morphants is reduced compared to wild-type) (Figure 3). Each segment within the distal tubule was moderately expanded, with a lengthened DE shown by expanded *slc12a1* expression, enlarged clusters of *stc1*-expressing cells that comprise the CS, and lengthened DL shown by *slc12a3* expression (Figure 3). The expression domain of *gata3* (PD) was also expanded (Figure 3). In contrast, the cloaca marker *aqp3* was unchanged (Figure 3A). These data suggest that RA signaling is necessary for podocyte formation and/or survival, as well as for establishing the normal pattern of nephron segmentation.

Multiple enzymes are capable of synthesizing RA and a recent analysis of the *neckless* (*nls*) (now called *aldh1a2*) mutant, which is defective in *raldh2*, demonstrated that there are additional sources of *raldh*-like enzyme activity in the zebrafish embryo [56–58]. Based on this, we hypothesized that the nephron phenotype in *raldh2* morphants represented the effect of reducing RA production, rather than a complete inhibition. To more fully block RA signaling, we utilized a competitive, reversible inhibitor of raldh enzymes, 4-diethylaminobenzaldehyde (DEAB) [59], which has been used to effectively prevent de novo RA synthesis in zebrafish embryos [58,60]. Wild-type embryos were treated with DEAB starting at 60% epiboly (early gastrula) until the 15 somite stage, and nephron segmentation was assayed at 48 hpf by double whole-mount in situ hybridization using *mhc* expression to mark the somites. Expression of the podocyte markers *wt1b*, *wt1a*, and *mafb* was absent in DEAB-treated embryos, suggesting that podocyte development was completely blocked (Figure 3 and unpublished data). Expression of the PCT and PST markers *slc20a1a* and *trpm7* was also absent, suggesting that these segments had failed to be specified (Figure 3). In contrast, *clck* expression (marking the DE and DL segments) was dramatically expanded, such that it was present throughout the entire tubule territory (Figure 3). These findings suggest that the pronephric tubule adopts a distal tubule identity when RA synthesis is inhibited. Within this ‘distal-only' pronephros, the DE marker *slc12a1* was expressed from the anterior limit of the tubule to almost the middle of its length, the DL marker *slc12a3* was expressed from the middle of the tubule to near its posterior limit, and the PD marker *gata3* was expanded by an additional four somite lengths (Figure 3). A marked expansion of *stc1*-expressing cells was also detected, with multiple clusters of cells arranged in bilateral stripes, as opposed to the small groups of *stc1*-expressing cells seen in wild-type embryos (Figure 3). Expression of *aqp3* was not altered, suggesting that progenitors of the cloaca are unaffected by the inhibition of RA production over this developmental interval (Figure 3). It is not known if the observed DEAB phenotype represents a full loss of RA signaling, as trace amounts of maternal RA have been detected in the yolk [61] and as zygotic *raldh2* transcripts are expressed prior to 60% epiboly [56,57]. Nevertheless, our results demonstrate that RA, produced by raldh2 and possibly one or more unknown raldh enzymes, plays an essential role in the formation of proximal nephron fates (podocytes, PCT, PST) and in suppressing the expansion of distal fates (DE, CS, DL, PD).

To determine more precisely when RA signaling was needed for the development of proximal segment fates, we performed a DEAB timecourse experiment (Figure 3). Blocking RA signaling from 90% epiboly (late gastrula) to the 5 somite stage caused a milder phenotype than the longer 60%- 15-somite exposure that was characterized by a loss of podocytes, reduced lengths of the PCT and PST segments, and small increases in the lengths of the distal segments (Figure 3). A slightly longer DEAB treatment, from 90% epiboly to the 10 somite stage, expanded distal segments further than the *raldh2* morphants or DEAB 90% epiboly-5 somite treated embryos (Figure 3). In addition, examination of the *slc20a1a* and *trpm7* expression patterns showed that the PST segment was ablated, while the PCT was only slightly shortened (Figure 3). Loss of PST identity was also confirmed by the absence of *slc13a1* and *slc22a6* transcripts, which are additional PST markers (Figure 2 and unpublished data). These findings suggest that, at least for this DEAB time window, the expansion of distal fates occurs at the expense of the PST segment. Finally, we tested whether DEAB treatment during somitogenesis would affect pronephros segmentation. DEAB treatment from 5–15 somites or 10–15 somites had no effect on the segmentation pattern of the pronephros (unpublished data), thus suggesting that an inhibition of RA signaling must be initiated prior to the 5 somite stage in order to affect nephron patterning. Taken together, our DEAB timecourse data indicate that RA signaling is required to induce proximal nephron fates and to prevent an expansion of distal fates, thereby establishing the normal pronephric segmentation pattern. Interestingly, our data suggest that different segments have different temporal requirements for RA signaling. RA is essential between 90% epiboly and the 5 somite stage to induce podocytes, between 90% epiboly and 10 somites to induce PST formation, and between 60% epiboly and 15 somites to form the PCT. The PCT segment is the most refractory to RA inhibition and is only lost following the longest DEAB treatment window (60% epiboly to 15 somites).

**Figure 4:**
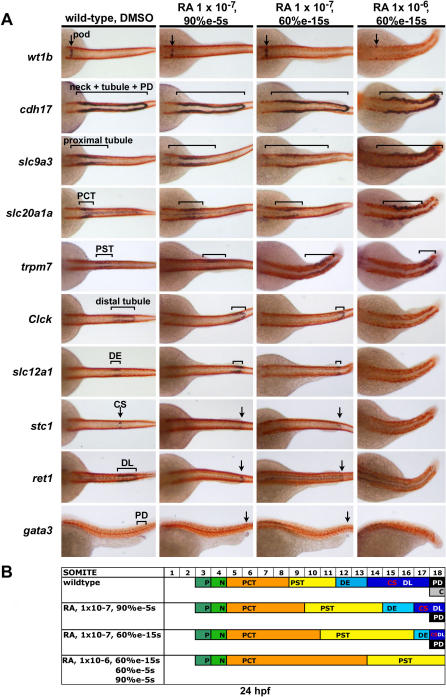
Exogenous RA Treatment Proximalizes the Pronephros (A) Expression of segment markers and *mhc* at 24 hpf in wild-type embryos incubated with either DMSO (control) or RA at the indicated dosage and times during development, by double whole-mount in situ hybridization. Brackets and arrows indicate expression domains. Abbreviations are: %e (percent epiboly), s (somite stage). Dorsal views are shown, with anterior to the left, with the exception of lateral views for *gata3* expression. (B) Summary of nephron segmentation with respect to embryo somite number for each RA experiment.

Given these findings, we tested whether increasing the concentration of RA by exogenous treatment would promote proximal nephron fates at the expense of distal fates. We exposed wild-type zebrafish embryos to RA during similar developmental intervals used for our DEAB experiments, and then assayed segment marker expression at 24 hpf by double in situ hybridization with *mhc* to mark the somites. Wild-type embryos treated with 1 × 10^−7^ M RA between 90% epiboly and 5 somites developed a normal number of podocytes, as evidenced by *wt1b* expression, but displayed expanded proximal tubule domains, shown by expression of *slc9a3* (marking both the PCT and PST), *slc20a1a* (PCT), and *trpm7* (PST) (Figure 4). Conversely, the *clck-*expressing distal tubule domain was reduced, due to reductions in the length of the DL (marked by *slc12a3)* and PD (marked by *gata3)* (Figure 4). However, the DE segment (marked by *slc12a1)* was unaffected (Figure 4). The position of the *stc1*-expressing CS segment cells was shifted posteriorly, and the level of expression was slightly reduced (Figure 4). A longer treatment window, from 60% epiboly–15 somites resulted in a more severe ‘proximalized' phenotype with a longer expanse of proximal tubule and a greater reduction in each distal tubule domain (Figure 4). In these embryos, the position of the *stc1*-expressing CS population was shifted even more posteriorly, and was located at the distal edge of the yolk sac extension (Figure 4A). We next treated wild-type embryos with higher dose of RA (1 × 10^−6^ M) over this same 60% epiboly–15 somites time window, as well as two shorter time periods: 60% epiboly–5 somites, and 90% epiboly–5 somites. Embryos treated for any of these time windows displayed a completely ‘proximalized' phenotype with the tubule domain being comprised entirely of proximal segment identities (Figure 4 and unpublished data). In these embryos, the proximal marker *slc9a3* was expressed throughout the *cdh17*-expressing tubule population (Figure 4 and unpublished data). Within this ‘proximal-only' pronephros, the PCT marker *slc20a1a* was expressed from the anterior limit of the tubule to somite 13, and the PST marker *trpm7* was expressed from somite 14 to the posterior limit of the tubule, where the *trpm7*-expressing tubules fused at the prospective site of the cloaca (Figure 4 and unpublished data). Expression of all distal segment markers was absent, suggesting that the DE, CS, DL, and PD had failed to be specified (Figure 4 and unpublished data). These results show that exogenous RA treatment from gastrulation stages until the 5 somite stage is sufficient to ‘proximalize' the pronephros, suggesting that this time period is the critical window when RA signaling is required for proximo-distal patterning of pronephric progenitors.

To further explore the notion that RA alters the patterning of renal progenitors prior to the 5 somite stage, we examined the expression patterns of the Notch ligands *deltaC* (*dlc*) and *jagged2a* (*jag2a*), and the renal transcription factors *wt1a*, *pax2a*, *pax8*, and *evi1*, as these genes are detected in the IM during early somitogenesis and have been implicated in early nephron patterning [44,62–64]. Wild-type embryos were treated with DMSO, DEAB, or 1 × 10^−6^ M RA from 60% epiboly until the 6 somite stage, and then IM gene expression was assayed by whole-mount double in situ hybridization together with *myoD* to mark the somites. Transcripts for *pax2a* and *pax8,* which label all pronephric progenitors, were found throughout the IM domain in a similar pattern in wild-type, DEAB-treated, and RA-treated embryos (Figure S5). Consistent with our previous results, *wt1a* expression was absent in DEAB-treated embryos, and *wt1a* was strongly up-regulated in RA-treated embryos (Figure S5). The expression domains of *deltaC* and *jag2a,* normally restricted to a proximal region of the IM adjacent to somites 2–5, were absent in DEAB-treated embryos, and expanded posteriorly in RA-treated embryos (Figure S5). Expression of *evi1*, found in the distal portion of the IM starting around somite 6, was shifted anteriorly to somite 3 following DEAB treatment and reduced to the most posterior group of IM cells following RA treatment (Figure S5). These findings demonstrate that changes in RA dosage during gastrulation to early somitogenesis are associated with gene expression changes in IM progenitors, far in advance of their mesenchymal-to-epithelial transition that creates the pronephric tubules.

### *cdx* Genes Establish the Position of the Pronephros along the A-P Axis

In addition to RA, multiple tissues along the embryonic axis are segmentally patterned by the *cdx* genes [25,34]. Loss of *cdx* gene function leads to an expansion of anterior trunk fates and axial elongation defects that result in a loss/truncation of the posterior trunk and tail [23–30,34]. In zebrafish, *cdx4^–/–^* mutant embryos display expanded *wt1a* expression at the 15-somite stage, suggesting that the podocyte lineage might be expanded and thus implicating *cdx* genes in pronephros patterning [24]. We therefore examined the formation of the pronephros in *cdx4^–/–^* and *cdx1a/4*-deficient (herein referred to as *cdx*-deficient) to assess development of the renal corpuscle, tubule, and pronephric duct. In addition, we used differential interference contrast (DIC) optics to visualize the somite boundaries and determine the size and position of each segment relative to the somites. It is important to note that the size of posterior somites in *cdx4^–/–^* embryos, and both the size and number of the somites in *cdx*-deficient embryos, is greatly reduced toward the posterior, due to the axial elongation defect [24,29–30]. A similar defect has also been observed in mouse *Cdx* mutants [34].

**Figure 5:**
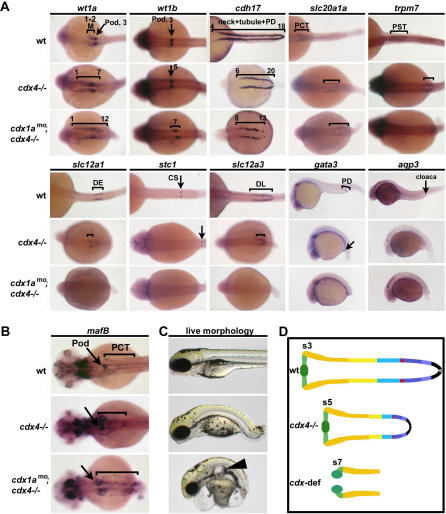
*cdx* Genes Position the Pronephros along the Embryonic Axis and Are Requisite for Distal Segment Formation Expression of segment markers was performed on wild-type, *cdx4^–/–^* mutants, and *cdx*-deficient embryos at (A) 26 hpf and (B) 48 hpf using whole-mount in situ hybridization. Brackets and arrows indicate expression domains in each embryo. Numbers indicate somite number. Dorsal views of embryos, with anterior to the left, with the exception of lateral views for *gata3* and *aqp3* expression. (C) Live wild-type, *cdx4^–/–^* mutants, and *cdx*-deficient embryos at 72 hpf. Arrowhead indicates glomerular cyst in the *cdx*-deficient embryo. Lateral views are shown, with anterior to the left. (D) Summary of nephron segmentation in *cdx4^–/–^* mutants and *cdx*-deficient embryos as compared to wild-type at 48 hpf. Numbers indicate anterior somite boundary of each pronephros.

At 24 hpf, wild-type embryos expressed *wt1a* in presumptive podocytes (located adjacent to somite 3, marked by an arrow in Figure 5A) as well as a population of presumptive mesenchymal cells located next to somites 1–3 in a broad lateral domain (indicated by a bracket and ‘M' in Figure 5A). *cdx4^–/–^* embryos had expanded *wt1a* expression at 24 hpf that ranged from a position anterior to somite 1 to approximately somite 7, and *cdx*-deficient embryos showed an even more dramatic posterior expansion that reached somite 12 (Figure 5A). As the expansion of *wt1a* in *cdx* mutant embryos could indicate increased numbers of podocytes and/or the mesenchymal population, we examined *wt1b* expression, which specifically marks podocytes [65]. Equivalent numbers of *wt1b*-expressing podocytes were formed in *cdx4^–/–^* mutant and wild-type embryos, but podocytes in *cdx4^–/–^* embryos were located more posteriorly, at the level of somite 5 (Figure 5A). *cdx*-deficient embryos developed a normal number of *wt1b-*expressing podocytes, though they were arranged in a pair of somewhat irregular linear groupings (rather than forming bilateral spherical clusters), and they were also located more posteriorly, adjacent to somite 7 (Figure 5A). We conclude from these observations that the loss of *cdx4* and *cdx1a/4* function progressively expands *wt1a* expression without increasing the number of podocytes and leads to podocyte formation at more posterior locations along the embryonic axis.

To assess tubule formation in *cdx* mutants, we examined the expression of *cdh17* at 24 hpf. While *cdx4^–/–^* embryos formed complete tubules that fused at the cloaca, *cdx*-deficient embryos displayed *cdh17* expression that was reduced and discontinuous, with the tubules failing to fuse (Figure 5A). Consistent with the posterior shifts in podocyte position, the A-P position of the tubule, marked by *cdh17* transcripts, was shifted caudally in both *cdx4^–/–^* and *cdx*-deficient embryos. In wild-type embryos, the tubule spans the length of somites 4–18, but was located from somites 6–20 in *cdx4^–/–^* embryos, and from somites 8–13 in *cdx*-deficient embryos (Figure 5A).

We next characterized the tubule segmentation pattern in *cdx* mutant embryos by examining the expression patterns of *slc20a1a* (PCT), *trpm7* (PST), *slc12a1* (DE), *stc1* (CS), *slc12a3* (DL), *gata3* (PD), and *aqp3* (cloaca). *cdx4^–/–^* embryos showed a slight expansion of the PCT, which spanned an additional two somites compared to wild-types (Figure 5A and unpublished data). The PST, DE, CS, and PD were all shorter than normal in *cdx4^–/–^* embryos, with the PD displaying the most severe reduction in length (Figure 5A and unpublished data). Transcripts for *aqp3* were not detected in *cdx4^–/–^* embryos, suggesting a defect in cloaca development (Figure 5A). These results indicate that loss of *cdx4* leads to a slight expansion in PCT fate with corresponding reductions in more distal fates.

In contrast to *cdx4^–/–^* embryos, *cdx*-deficient embryos showed discontinuous *slc20a1a* expression and failed to express *trpm7*, *slc12a1*, *stc1*, *slc12a3*, *gata3,* or *aqp3* (Figure 5A). These results suggest that the tubule territory in *cdx*-deficient embryos acquires a PCT identity, while the remaining nephron segments fail to develop. These defects were not the result of delayed development, as at later developmental stages, *cdx*-deficient embryos continued to possess tubules that only expressed PCT-markers, and the tubules never fused caudally (unpublished data). In addition, expression analysis of the podocyte marker *mafB* in *cdx*-deficient embryos at 48 hpf revealed that podocytes fail to fuse into a single renal corpuscle. Instead, the podocytes formed bilateral corpuscles that were dilated compared to wild-type and *cdx4^–/–^* embryos, presumably due to fluid accumulation (Figure 5B). Consistent with this, *cdx*-deficient embryos had developed glomerular cysts by 72 hpf, as well as severe pericardial edema, indicative of renal failure (Figure 5C). In contrast, *cdx4^–/–^* embryos never exhibited glomerular cyst formation or edema, suggesting that although various segment lengths were shortened, these embryos were able to maintain adequate kidney function (Figure 5C).

In summary, our expression analyses show that *cdx* deficiency causes a posterior shift in the location of the pronephros along the embryonic axis (Figure 5D). While the podocytes and PCT populations formed relatively normally, the PST and distal tubule segments were reduced or absent in *cdx4^–/–^* and *cdx*-deficient embryos, respectively (Figure 5D). Thus the *cdx* genes, acting either directly or indirectly, are required for the formation of the distal nephron segments and establishing the normal segmentation pattern of the pronephros.

### *cdx* Genes Mediate the Expression of RA Synthesizing and Degrading Enzymes

**Figure 6:**
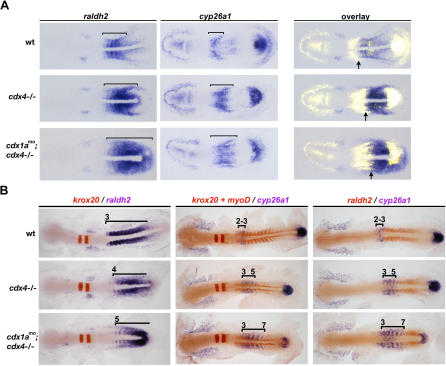
*cdx* Genes Regulate the Expression Boundaries of *raldh2* and *cyp26a1* (A) Expression of *raldh2* and *cyp26a1* in wild-type, *cdx4^–/–^* mutants, and *cdx*-deficient embryos by whole-mount in situ hybridization. Brackets indicate *raldh2* and *cyp26a1* expression domains in the anterior paraxial mesoderm of 5 somite stage embryos. Right column is an overlay of the *raldh2* and *cyp26a1* expression patterns, arrows indicate the anterior boundary of the presumptive RA source. (B) Double whole-mount in situ hybridization showing co-staining of *krox20*, *myoD*, *raldh2*, and *cyp26a1* at 10 somites in wild-type, *cdx4^–/–^* mutants, and *cdx*-deficient embryos (gene names are color-coded to match their staining product). Brackets and numbers indicate *raldh2* and *cyp26a1* expression domains and somite position, respectively

Given the largely opposite effects of *cdx*-deficiency and loss of RA signaling on nephron patterning, as well as the recent report that *cdx* genes control how the hindbrain responds to RA during its patterning [30,31], we wondered if an interplay between these pathways was operative during pronephros segmentation. The location and level of RA within tissues is dependent on the expression of *raldh-*synthesizing enzymes and *cyp26-*degrading enzymes [15,54]. We therefore investigated the expression of these genes in *cdx*-deficient embryos during early somitogenesis, as our previous experiments suggested that the IM is being influenced by RA signaling at this time. At the 5 somite stage, expression of *raldh2* in the paraxial mesoderm was expanded posteriorly in *cdx4^–/–^* embryos compared to wild-types (Figure 6A). An even greater posterior expansion was seen in *cdx*-deficient embryos, with *raldh2* transcripts being detected throughout the entire unsegmented paraxial mesoderm and tailbud region (Figure 6A). Expression of the RA-catabolizing enzyme *cyp26a1* in the upper trunk region was also expanded posteriorly in *cdx4^–/–^* embryos at the 5 somite stage, and more extensively expanded in *cdx*-deficient embryos (Figure 6A). To visualize how the combined changes in *raldh2* and *cyp26a1* expression altered the source of RA along the trunk, we generated digital overlays of these expression patterns. This analysis suggested that the anterior boundary of RA production (i.e., the junction of the *cyp26a1* and *raldh2* expression domains) was located more posteriorly in *cdx4^–/–^* mutants compared to wild-types, and that this posterior shift was more pronounced in *cdx*-deficient embryos (arrows in Figure 6A).

To better quantitate these posterior shifts, we examined expression of *raldh2* and *cyp26a1* at the 10 somite stage when the somites could be visualized by staining for *myoD* transcripts. We found that the *raldh2* expression boundary in *cdx4^–/–^* and *cdx*-deficient embryos was shifted posteriorly by 1 and 2 somites, respectively, compared to wild-type embryos (Figure 6B). The *cyp26a1* domain in wild-type embryos occupied the region of somites 2–3, just rostral and slightly overlapping with *raldh2*, as shown by double in situ hybridization (Figure 6B). In *cdx4^–/–^* embryos, *cyp26a1* transcripts were detected in the region of somites 3–5, whereas in *cdx*-deficient embryos they extended from somites 3–7 (Figure 6B). These analyses reveal a striking correlation between the presumptive source of RA at the 10 somite stage and the axial position of the pronephros at 24 hpf in each genotype (i.e., the source of RA and the position of the pronephros both start at somites 3, 5, and 7 in wild-type, *cdx4^–/–^*, and *cdx*-deficient embryos, respectively). The combination of these data, together with the results from our DEAB experiments, suggest a model in which the *cdx* genes act upstream of *raldh2* and *cyp26a1* to localize the source of RA along the A-P axis, and that RA, in turn, acts on the IM to induce the proximal segments and prevent an expansion of the distal segment fates.

### Inhibition of RA Synthesis Is Sufficient to Rescue Distal Segment Formation in *cdx* Mutants

**Figure 7:**
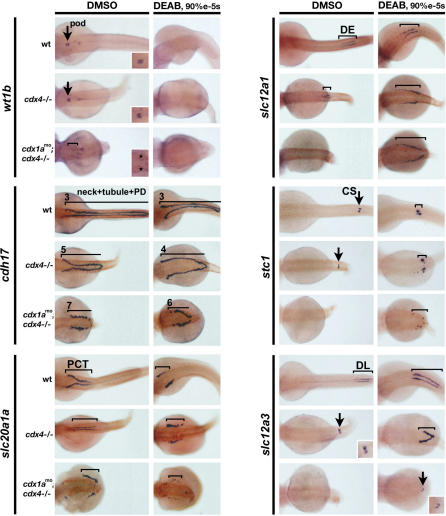
Blocking RA Production in *cdx* Mutants Partially Rescues Pronephros Position and Distal Segment Formation (A–C) Expression of *cdh17*, *slc20a1a*, and *slc12a1* at 48 hpf in wild-type, *cdx4^–/–^* mutants, and *cdx*-deficient embryos that were treated with DMSO (control) or DEAB from 90% epiboly to the 5 somite stage. Lines and numbers indicate expression domains and somite position, respectively. Arrows indicate podocyte and CS positions. Dorsal views are shown, with anterior to the left. Insets show enlarged dorsal views of podocyte (asterisk marks cystic glomeruli which stain weakly) and DL segment staining.

If our model is correct, then inhibiting RA synthesis in *cdx*-deficient embryos should rescue pronephric positioning and the formation of the distal tubule segments. To test this, we treated *cdx4^–/–^* and *cdx*-deficient embryos with DEAB from 90% epiboly to the 5-somite stage and examined pronephros segmentation. In support of our model, we found that *cdx4^–/–^* and *cdx*-deficient embryos exhibited a one-somite anterior shift in the position of the pronephros, as shown by expression of *cdh17* (Figure 7). In addition, the development of podocytes was abrogated, and the length of the PCT was reduced in DEAB-treated *cdx4^–/–^* and *cdx*-deficient embryos (Figure 7), consistent with our findings in wild-type embryos (Figure 3). We observed that the DE segment was increased in DEAB-treated *cdx4^–/–^* embryos, as shown by an expansion of the *slc12a1* expression domain (Figure 7). DEAB treatment also increased the number of CS cells in *cdx4^–/–^* mutants, shown by the expression of *stc1*, and increased the DL segment length, evidenced by expansion of the *slc12a3*, *romk2*, and *clck* expression domains (Figure 7 and unpublished data). In *cdx*-deficient embryos treated with DEAB, formation of the DE, CS, and DL segments was rescued, shown by expression of *slc12a1*, *stc1*, and *slc12a3*, respectively (Figure 7). A similar rescue of the expression of the DE and DL markers *romk2* and *clck* was also observed (unpublished data). These findings demonstrate that *cdx* gene function is not necessary to specify distal segment identity directly, but instead suggests that the abrogation of distal segment formation in *cdx*-deficient mutants is related to the level of RA that the renal progenitors are exposed to. Taken together with the above results, these finding provide good evidence that the pronephric positioning defect and failure to form the distal tubule identities in *cdx*-deficient embryos is caused by mis-localization of the RA source along the A-P axis.

## Discussion

The segmentation of the nephron into distinct functional units is critical for kidney function. How this proximodistal pattern is established during renal development is poorly understood. In this study, we show that the zebrafish pronephros is an excellent model to study nephron segmentation, as it is comprised of extensive proximal and distal tubule segments, rather than mostly duct as is currently believed. By investigating the effects of RA and *cdx* gene activity, we uncovered a role for RA in the formation of proximal nephron fates, and found that *cdx*-deficient animals lacked distal nephron fates, concomitant with a posterior shift in the position of the pronephros. An analysis of the *raldh2* and *cyp26a1* expression domains in *cdx*-deficient embryos revealed a correlation between the presumptive source of RA along the trunk and the A-P position of the pronephros, thus suggesting that the *cdx* genes determine where the kidney forms by localizing the production of RA along the axis. Consistent with this, we found that inhibiting RA synthesis in *cdx* deficient embryos partially rescued the pronephric positioning defect and restored expression of distal tubule markers.

### The Role of RA in Pronephros Segmentation

Retinoid signaling plays essential roles in the A-P patterning of a number of diverse tissues in the embryo. During early development, a source of RA in the upper trunk (cervical) region is produced by the action of the RA synthetic enzyme, Raldh2, which is expressed in the anterior paraxial mesoderm [54]. The coordinate expression of RA-catabolizing Cyp26 enzymes in surrounding tissues creates a so-called ‘sink' for this RA source [54]. Collectively, these enzymes are thought to create a gradient of RA activity that diffuses into surrounding tissues [34,54]. The functions of this RA source have been extensively studied in the developing hindbrain, where the effects of graded RA signaling are thought to create nested expression domains of RA-responsive genes that drive A-P segmentation of the hindbrain into a series of rhombomeres [15,54]. In addition to regionalizing the overlying neurectoderm, RA produced in the upper trunk paraxial mesoderm has been implicated in the regionalization of the underlying endoderm. Studies in zebrafish have shown that RA acts directly on the endoderm to specify hepatopancreatic progenitors that give rise to the liver and pancreas [17,18]. RA also influences mesodermal cell fate decisions during zebrafish development, including the formation of the pectoral fin field—which arises from the lateral plate mesoderm adjacent to the upper trunk somites [56,57,66]—and the heart [67]. In the latter case, inhibition of RA synthesis leads to an expansion of precardiac mesoderm, resulting in an excessive number of myocardial progenitors [67]. These findings indicate that, in addition to acting as an inducer of cell fates such as in the hindbrain and endoderm, RA also plays an important role in restricting certain cell fates.

**Figure 8:**
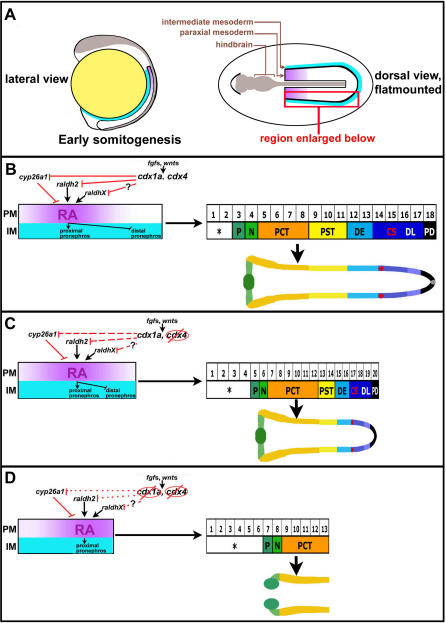
Model for How the *cdx* Genes and RA Establish Pronephros Position and Segmentation (A) At the end of gastrulation, the IM is located lateral to the PM. The production of RA in the PM is localized to the anterior-most region (purple). RA is proposed to diffuse as a gradient across to the adjacent IM such that the anterior IM is exposed to high RA levels, which induce proximal fates and suppress expansion of distal fates. (B) In wild-type embryos, *cdx1a* and *cdx4* set the expression boundaries of *raldh2* (and potentially other putative *raldh*-like genes, *'raldhX'*) and *cyp26a1*, thereby restricting RA production to the anterior PM. The absence of *cdx4* function (C) and *cdx1a/4* function (D) causes posterior shifts in the RA source, due to the combined posterior shifts of both the *raldh2* and *cyp26a1* expression domains. These shifts in the presumptive RA zone lead to pronephros formation at a more posterior location, and an expansion of anterior intermediate mesodermal fates (indicated by an asterisk). In *cdx1a/4*-deficient embryos, the posterior shift of the pronephros, together with the body truncation, results in an abrogation of distal nephron segments.

Our study now adds the IM as another mesodermal derivative that is patterned by RA. Our results show that RA production is essential during gastrulation and early somitogenesis for the induction of proximal nephron fates as well as to restrict the expansion of distal nephron fates. Over this period of development, RA is produced by the anterior paraxial mesoderm (PM). The IM, which gives rise to the pronephros, is located lateral to the PM (Figure 8). Given the role of RA as a diffusible morphogen in other tissues, we hypothesize that RA diffuses from the PM and establishes a gradient along the IM, with high levels of RA inducing proximal fates and low RA levels being permissive for distal fates (Figure 8). Our time-course experiments with DEAB support this view, with the most severe reduction in proximal fates (and concomitant expansion in distal segments) corresponding to the longest treatment window. However, further work is needed to determine the nature of the RA gradient, as well as how dynamic fluctuations in retinoid availability [54] affect the dose and length of time that the renal progenitors are exposed to RA. A gradient-free model has recently been proposed for RA-dependent hindbrain patterning, based on the finding that sequential expression domains of the *cyp26a1*, *cyp26b1*, and *cyp26c1* genes are essential for rhombomere boundary establishment [68,69]. It is unclear if a similar mechanism might operate during pronephros segmentation, as *cyp26b1* and *cyp26c1* do not show a nested pattern of expression in the IM.

Overall, the effects of RA on the patterning of the IM can be regarded as ‘anteriorizing.' Our finding that exogenous RA treatment induces proximal tubule fates to form throughout the pronephros supports this conclusion. Classically, RA is known as a ‘posteriorizing' factor due to its effects on the central nervous system, where an inhibition of RA signaling causes an expansion of anterior neural fates in the hindbrain [15,54]. Thus we conclude that RA can actually have both anteriorizing and posteriorizing activities, depending on the tissue in question. A unified way to characterize these effects would be to consider the upper trunk RA source as an organizing center, akin to the dorsal organizer in the gastrula, that locally patterns cell types in all three germ layers.

### Additional Roles of RA in Kidney Development

Previous studies have implicated RA as a regulator of renal development. Animal cap experiments in *Xenopus* showed that RA, together with Activin, is sufficient to induce the formation of pronephric tubules [70]. A more recent study in *Xenopus* reported that overexpressing various RA antagonists results in a complete loss of the pronephros (glomus, tubules, and duct) [55]. This phenotype is more severe than what we observed and may reflect differences in the efficacy of DEAB to completely block RA production compared with other RA antagonists. The pronephric tubules in *Xenopus* are segmented into proximal and distal segments [48], similar to the zebrafish pronephros, however a role for RA in *Xenopus* nephron segmentation has not been reported.

In mammals, it has long been known that vitamin A deficiency causes severe renal malformations [71]. Targeted mutagenesis of the *RAR* genes in mouse, followed by elegant rescue experiments, established an important role for RA as a dose-dependent inducer of the GDNF receptor *Ret* [72,73]. GDNF is an essential regulator of ureteric bud branching morphogenesis, and loss of GDNF signaling results most frequently in renal agenesis [74]. Because ureteric bud branching is an essential prerequisite for nephrogenesis, RA serves a key role in stimulating nephron formation. At present it is not known whether RA is also involved in the proximodistal patterning of metanephric nephrons. Interestingly, *Raldh2* transcripts are found in podocyte progenitors, whereas *Cyp26a1* is expressed by the tubule anlagen during metanephros development, suggestive of a role for RA in mammalian nephron patterning [75].

### Downstream Targets of RA during Nephron Segmentation

Transplantation studies in frogs suggest that RA may act directly on pronephric precursors [55]. However, the downstream targets of RA in the IM are not known. In the hindbrain, the presumptive RA gradient is thought to regulate rhombomere segmentation by activating the expression of the anterior, 3' *Hox* genes, i.e., those comprising the 1st through 5th paralog groups [15,54,76]. In zebrafish, transcripts for *hoxb1a*, *hoxb1b*, and *hoxb5a* are found in proximal portions of the IM, thus making them potential candidates for mediating the effects of RA during pronephros segmentation [34]. Future studies using single, double, and triple morpholino injections can test the importance of these *hox* genes for renal development.

The effects of RA on pronephric segmentation may also be coordinated by the action of non-Hox pathways. Our results suggest the intriguing possibility that RA signaling targets may include renal transcription factors as well as members of the Notch signaling pathway. The gene *evi1* encodes a zinc-finger transcription factor that has been implicated in patterning distal regions of the pronephros in *Xenopus*, and overexpression of *evi1* was found to inhibit proximal segment formation [63]. These data are consistent with our results showing that expression of *evi1* in the IM was expanded following DEAB treatment, and reduced following exposure to exogenous RA. Another renal transcription factor candidate is the *odd-skipped related transcription factor 1* (*osr1*) encoding a zinc-finger repressor. Recent studies in *Xenopus* and zebrafish have shown that *osr1* is expressed in the ventral mesoderm during gastrulation and later in an anterior domain of the IM [77]. Morpholino-mediated knock-down of *osr1* leads to defects in the formation of podocytes and proximal tubule progenitors [77], consistent with Osr1 participating in a common pathway with RA. The Notch pathway may also interact with RA during nephron segmentation. Conditional knockout of *Notch2* in the mouse metanephros results in a loss of podocytes and proximal tubule fates, whereas distal markers are relatively unaffected [78]. In zebrafish, Notch signaling has been shown to regulate the differentiation of multiciliated cells and principle cells in the pronephric tubules [44,45]. However, a role for Notch signaling in the formation of proximal nephron fates is also suggested by the expression pattern of the Notch ligands *deltaC*, *jag1b*, and *jag2a*, which are restricted to proximal portions of the intermediate mesoderm [62,64]. Simultaneous knockdown of *jag1b/2a* results in an abnormally small renal corpuscle and dysmorphic proximal tubules, consistent with a conserved role for Notch signaling in proximal nephron development [79]. Our finding that DEAB treatment abrogates *deltaC* and *jag2a* expression in the proximal IM, while exogenous RA expands their expression, supports a role for RA acting upstream of the Notch pathway.

### *cdx* Genes Play an Integral Role in Positioning the Axial Location of RA

Our study provides evidence that *cdx* genes control the expression domains of *raldh2* and *cyp26a1* along the embryonic axis. The boundaries of both *raldh2* and *cyp26a1* are progressively shifted toward the posterior in *cdx4* and *cdx1a/4*-deficient embryos, suggesting that the upper trunk source of RA is posteriorly shifted. We hypothesize that this posterior shift in RA production results in a posterior shift in the position of the pronephros (Figure 8). We propose that this effect, combined with the axial elongation defects, leads to reduced or absent distal segment fates. The ability to rescue distal segments by treating *cdx* mutants with a pulse of DEAB is consistent with this model, and also demonstrates that *cdx* function is not requisite for the induction of distal fates from the intermediate mesoderm. Thus additional, as yet unidentified pathways, are responsible for directing distal fates. While our data supports the notion that Cdx factors exert their effects by the regulation of RA signaling, it does not rule out the possibility that Cdx factors may also function to repress proximal fates independent of RA signaling.

Given the mounting evidence that the upper trunk RA source is an important organizing center, we would predict that both the patterning and positioning of numerous organs would be affected in *cdx* mutants. Consistent with this, defects in several mesodermal fates that arise in the anterior trunk region have been observed in *cdx-*deficient embryos. Vascular precursors are progressively expanded when *cdx* activity is abrogated, and blood precursors are both reduced and shifted posteriorly in *cdx* mutants [24,29]. In addition to mesodermal defects, *cdx* mutants also display patterning defects in the neurectoderm that gives rise to the anterior spinal cord [30,31]. We hypothesize that many, if not all, of these defects in *cdx* mutants are caused by the abnormal localization of RA along the A-P axis.

The loss of *cdx* gene function in both zebrafish and murine models has been shown to cause global shifts in *hox* gene expression in the mesoderm and neurectoderm [24,25,29–30,34]. Given the rostral shifts and expansions of both *raldh2* and *cyp26a1* expression observed in *cdx4* and *cdx1a/4*-deficient embryos, Hox transcription factors are attractive molecules for regulating *raldh2* and *cyp26a1* expression. Defects in blood formation in *cdx4*-null zebrafish can be rescued by the overexpression of several *hox* genes [24], and the overexpression of *hoxa9a* also results in a partial rescue of the axis elongation defect in *cdx4^–/–^* embryos [29]. Future studies are needed to examine whether *hox* gene overexpression(s) can rescue pronephros positioning and formation of distal segments in *cdx* mutant embryos.

In conclusion, our studies have revealed an important link between the *cdx* genes and localization of RA, and provide evidence that RA signaling is a central determinant of pronephros A-P segmentation. Our results establish the zebrafish embryo as a simplified model of vertebrate nephron segmentation that will further our understanding of mammalian nephron segmentation, and provide insights into the causes of kidney birth defects and renal disease in humans.

## Materials and Methods

### Zebrafish husbandry, genetic strains, chemical treatments, and morpholinos.

Zebrafish were maintained and staged as described [80,81]. Tübingen strain wild-type embryos were used for all experiments. DEAB and all-trans retinoic acid (Sigma-Aldrich) were dissolved in 100% dimethyl sulfoxide (DMSO) to make a 1 M stock and aliquots were stored at −80°C. For DEAB and RA treatments: embryos were incubated in 1.6 × 10^−5^ M DEAB/DMSO in E3 embryo media, 1 × 10^−6^ M or 1 × 10^−7^ M RA/DMSO in E3 embryo media, or 1.6 × 10^−5^ M DMSO (control) in E3 in the dark over particular developmental intervals, then washed five times with E3 and then fixed at 24 or 48 hpf. These experimental treatments were fully penetrant and produced consistent results at the doses and treatment windows that were examined. *raldh2* morpholino (CAACTTCACTGGAGGTCATCGCGTC) was injected into 1-cell wild-type embryos. Incrosses of *kgg^tv205^* heterozygous adults (maintained on the Tübingen strain) were used to obtain *cdx4^–/–^* embryos and were injected at the 1-cell stage with *cdx1a* morpholino (CAGCAGATAGCTCACGGACATTTTC) as described [29] to obtain *cdx*-deficient embryos. Both *raldh2* and *cdx1a* morpholinos produced fully penetrant effects. Embryos were raised to appropriate stages and fixed in 4% paraformaldehyde (PFA)/1×PBST for gene expression analysis. For all reported gene expressions, at least 20 embryos were examined.

### Zebrafish in situ hybridization.

Whole-mount in situ hybridization of zebrafish embryos was performed as previously described [24]. The expression patterns of *cdh17*, *clck*, *cyp26a1*, *evi1*, *gata3*, *mhc*, *myoD*, *nbc1*, *pax2a*, *pdzk1*, *raldh2*, *ret1*, *sall1*, *sglt1*, *slc4a2*, *slc20a1a*, *wt1a*, and *wt1b* were previously reported [14,24,29,37,55,56,61,82–86]. For antisense probe production, we used the following IMAGE clone template plasmids, restriction enzymes for DNA linearization, and RNA enzymes: *mafb*: 7995399, pExpress-1, EcoR1, T7; *rfx2*, pBK-CMV, template was PCR amplified using primers GTGAATTGTAATACGACTCACTATAGGG and TTAACCCTCACTAAAGGGAACAAA, T7; *slc9a3*: 6996791, pExpress-1, EcoRI, T7; *slc26a2*: 4760214, pBK-CMV, EcoRI, T7; *slc13a1*: 6793065, EcoRV, t7; *slc13a3*: 4744276, pCMV-sport6.1ccdb, EcoRI, T7; *slc22a6*: 4744276, pBK-CMV, SalI, T7; *slc12a1*: pBK-CMV, EcoRI, T7; *slc12a3*: 7037010, pExpress-1, EcoRI, T7; *stc1* was amplified from 24 hpf embryo cDNA using primers ATGCTCCTGAAAAGCGGATTT and TTAAGGACTTCCCACGATGGA and cloned into pGemTEasy, NcoI, Sp6.

### Murine gene cloning and in situ hybridization.

Gene-specific primers spanning 700–1,000 bp of the coding sequence were used to amplify DNA fragments from E15.5/P0 kidney cDNA pools, and the PCR products of the right size were cloned into the pCRII-Topo vector (primer sequences available upon request). DNA templates for riboprobe production were generated by PCR with T7 and Sp6 Ready Made primers (Integrated DNA Technologies) from PCRII-TOPO clones or T7 and T3 Ready Made primers from Bmap library clones. Digoxigenin-labeled anti-sense riboprobes were synthesized from the PCR product and purified with Micro Bio-spin columns P-30 Tris RNase-free (Bio-Rad). Probes were diluted with prehybridization buffer (50% formamide, 5×SSC, pH4.5, 50 μg/ml yeast tRNA, 1% SDS, 50 μg/ml heparin) to 10 μg/ml and stored at −80 °C. Neonatal kidneys were dissected free of surrounding tissues except the ureter and fixed with 4% PFA at 4 °C for 24 h. After PBS washes, they were incubated with 30% sucrose at 4 °C overnight. Kidneys were swirled in five dishes of OCT to remove sucrose and mounted in OCT in a dry ice/ethanol bath. The OCT blocks were stored at −80 °C. Sections were cut at 20 μm and air dried. Sections were post-fixed with 4% PFA for 10 min, treated with 10 μg/ml proteinase K for 10 min and post-fixed for 5 min. Slides were acetylated (1.33% Triethanolamine, 0.065% HCl, 0.375% acetic anhydride) for 10 min and dehydrated with 70% ethanol and 95% ethanol for 5 min each. Slides were air dried then incubated with 500 ng/ml digoxigenin-labeled riboprobes at 68 °C overnight. Hybridized sections were washed with 50% formamide, 1×SSC, pH4.5 for 30 min at 65 °C, treated with 2 μg/ml RNase for 15 min at 37 °C, and washed with 2×SSC, pH4.5 for 30 min, and twice with 0.2×SSC, pH4.5 for 30 min at 65 °C. Slides were washed three times at room temperature with 1×MBST (0.1 M maleic acid, 0.15 M NaCl, 0.1% Tween-20, pH7.5) for 5 min each, and incubated with blocking solution (2% Boehringer Mannheim (BM) blocking reagent) in 1×MBST, 20% heat-inactivated sheep serum) for 1 h. After incubation with anti-digoxigenin antibody-AP (Roche, 1:4000) at 4 °C overnight, sections were washed with 1×MBST at room temperature, 5 min for three times, then with NTMT (0.1 M NaCl, 0.1 M Tris-HCl, pH9.5, 50 mM Mg_2_Cl, 0.1% Tween-20, 2 mM Levimasole) for 10 min, and developed with BM purple (Roche). Color reactions were stopped with fixatives (4% PFA, 0.2% glutaraldehyde) and sections mounted with glycergel mounting media (DAKO). Images were captured with a Nikon DXM1200 digital camera attached to a Leitz DMRB microscope.

## Supporting Information

Figure S1 Zebrafish Proximal Pronephros Segment Domains at 24 hpf Double in situ hybridizations for segment-restricted genes (purple) and *mhc* (red).
(2.3 MB JPG)

Figure S2 Zebrafish Distal Pronephros Segment Domains at 24 hpf Double in situ hybridizations for segment-restricted genes (purple) and *mhc* (red).
(2.4 MB JPG)

Figure S3 Zebrafish Proximal Pronephros Segment Domains at 48 hpf Double in situ hybridizations for segment-restricted genes (purple) and *mhc* (red).
(1.7 MB JPG)

Figure S4 Zebrafish Distal Pronephros Domains at 48 hpf Double in situ hybridizations for segment-restricted genes (purple) and *mhc* (red).
(1.5 MB JPG)

Figure S5 Changes in RA Signaling Cause Early Alterations in Gene Expression Throughout the Intermediate Mesoderm Expression of intermediate mesodermal markers (purple) and *myoD* (red) at the 6 somite stage in wild-type embryos incubated with DMSO, DEAB, or 1 × 10^−6^ M RA from 60% epiboly until the stages shown, by double in situ hybridization. Brackets indicate expression domains, and numbers indicate somite identity.
(1.0 MB JPG)

## Abbreviations

CS: corpuscle of Stannius
DE: distal early
DEAB: 4-diethylaminobenzaldehyde
DL: distal late
dpf: days post-fertilization
hpf: hours post-fertilization
IM: intermediate mesoderm
PCT: proximal convoluted segment
PD: pronephric duct
PST: proximal straight segment
PM: paraxial mesoderm
RA: retinoic acid
